# Normal Heart Ventricular Tachycardia Associated with Pregnancy: Successful Treatment with Catheter Ablation

**DOI:** 10.1016/s0972-6292(16)30733-1

**Published:** 2014-03-12

**Authors:** Andrew J Hogarth, Lee N Graham

**Affiliations:** Department of Cardiac Electrophysiology, The Yorkshire Heart Centre, Leeds General Infirmary, Leeds, UK

**Keywords:** Ventricular tachycardia, Right ventricular outflow tract, Pregnancy, Catheter ablation, Verapamil

## Abstract

**Background:**

Normal heart ventricular arrhythmia occurring during pregnancy has been previously described. Whilst there are established reports of catheter ablation to treat supraventricular arrhythmia during pregnancy, there are no reports of ablation to treat ventricular tachycardia.

**Case:**

We present the case of a 36 year old women, 31 weeks into an otherwise uncomplicated pregnancy, experiencing significant, troublesome and drug refractory tachycardia emanating from the right ventricular outflow tract.

**Conclusion:**

We describe a successful radio frequency ablation in the third trimester of pregnancy.

## Case Report

A 36-year old woman was admitted acutely to our institution with a four week history of increasingly intrusive palpitations associated with pre-syncope. She was 31 weeks into her second pregnancy having experienced no problems during the first or second trimester or at all during her previous pregnancy 6 years earlier. Ambulatory monitoring organised prior to her admission had demonstrated frequent unifocal premature ventricular contractions (PVC) including couplets and triplets, and short salvos of non-sustained ventricular tachycardia (VT) corresponding with symptoms. The initial physical examination, electrolytes and laboratory investigations were unremarkable. Obstetric assessment revealed a healthy fetus consistent with 31 weeks of gestation.

**Figure 1:**
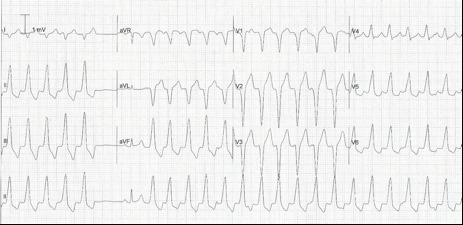
12 lead ECG (25mm/second sweep) during an episode of typical symptoms. Bursts of broad complex tachycardia are seen with a left bundle branch block morphology and precordial transition at V4 consistent with origin form the right ventricular outflow tract. Dissociated atrial activity (p waves) can be seen (arrow). The 6th complex along from the left is a normal sinus beat. Such bursts of tachycardia are characteristic of normal heart RVOT VT.

Her electrocardiogram during a typical episode revealed a monomorphic wide-complex tachycardia of left bundle branch block morphology consistent with an origin from the right ventricular outflow tract (Figure 1). Echocardiography demonstrated a mildly dilated left ventricle with normal function and an ejection fraction of 63%. Initial attempts to control her arrhythmia medically with oral and intravenous metoprolol were unsuccessful. The patient was subsequently treated with oral verapamil therapy but, despite initial benefit, suffered with recurrent and increasingly sustained episodes of VT associated with hypotension and pre-syncope. Following obstetric consultation, the option of catheter ablation was offered as a therapeutic option. After discussion of the potential risks to the mother and foetus, both of the procedure and of potential radiation exposure, informed consent was obtained.

 The patient was brought to the electrophysiology laboratory in the postabsorptive state and placed in the left lateral position using a wedge to decompress the inferior vena cava. A midwife was present to monitor the foetal heart rate. Lead shielding of the abdomen and pelvis was used to shield the fetus as much as possible if any fluoroscopic radiation was needed. Venous access was obtained from the right femoral vein. Although we intended to perform the procedure with zero fluoroscopy, owing to the distortion of the pelvic anatomy by the gravid uterus and the tilt from the wedge, minimal fluoroscopy (60 seconds, 9.9mGy) was utilised to position the catheters in the heart. Catheters were placed in the right ventricular apex and coronary sinus.

**Figure 2:**
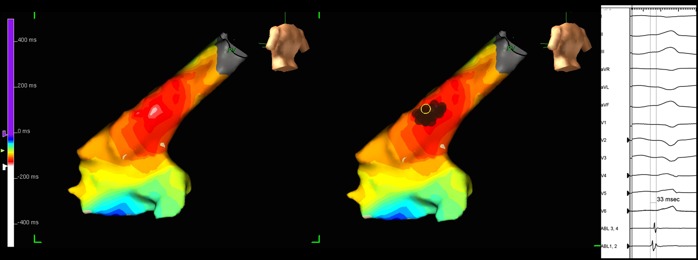
A 3-dimensional geometry of the RV outflow tract using the NavX system (St Jude Medical). The left hand image shows an isochronal map of local electrical activation time in relation to the surface QRS (red to white is the earliest local activation suggestive of the endocardial breakout of the arrhythmia). The next image shows the area of ablation as represented on the NavX system (red dots). The right hand panel shows the local electrical signal on the ablation catheter at the point where subsequent ablation abolished tachycardia (top to bottom surface ECG leads, ablation catheter proximal to distal electrode pairs. Sweep speed 100mm/second).

 The remainder of the procedure was performed with zero-fluoroscopy using a 3-dimensional mapping system to create a geometrical shell of the right ventricular outflow tract (NavX, St. Jude Medical, St Paul, Minneapolis, USA). During frequent periods of non-sustained VT earliest endocardial activation was localised to the high outflow tract (Figure 2). At this location the local electrogram on the ablation catheter preceded the onset of the QRS complex by 33ms (Figure 2). Catheter tip pressure at this site induced sustained VT with subsequent radiofrequency ablation terminating VT. There was no recurrence during a 30-minute period of observation. The procedure was uncomplicated and no further PVCs or VT were observed on telemetry over the following 48-hours.

 The patient remained asymptomatic and interim ambulatory monitoring after 8 weeks demonstrated no evidence of recurrent arrhythmia. She delivered a healthy child at term by elective caesarean section without complication.

## Comment

We report a case of new onset right ventricular outflow tract tachycardia during the third trimester of pregnancy in an otherwise healthy young female. Catheter ablation was successfully performed after failure of conservative treatment with antiarrhythmic drugs.

Pregnancy is associated with an increased risk of arrhythmia in women with both structurally normal and abnormal hearts. Furthermore, in women with a prior history of arrhythmia, exacerbations during pregnancy are frequent and have a negative impact on fetal and neonatal outcome mostly due to a higher incidence of premature labour.[1] It is unknown whether this relates to the arrhythmia itself or due to concomitant use of antiarrhythmic drugs or anticoagulants.

Factors predisposing to the development or exacerbation of arrhythmia during pregnancy include significant hemodynamic changes, hormonal flux, changes in autonomic tone, increased sensitivity to circulating catecholamines, hypokalaemia of pregnancy, and presence of structural heart disease.[2]

There are few reports of new-onset VT during pregnancy in the absence of structural heart disease.[2] A previous report of 11 pregnant women with new-onset VT suggested an equal distribution over the three trimesters with over 70% of arrhythmias originating from the right ventricular outflow tract.[2] The mechanism of VT initiation in pregnancy has not been determined but is unlikely to be simply related to circulating oestrogen concentrations since arrhythmia precipitation has been shown to be associated with both low oestrogen states, such as the menopause or pre-menstrually, as well as high oestrogen states such as pregnancy. Furthermore, oestrogen has been shown to demonstrate calcium antagonistic properties in cardiac myocytes which might suggest an antiarrhythmic effect. Consideration should also be given to the potential arrythmogenic milieu created by an imbalance between dominant progesterone and oestrogen. There have been reports of pregnancy related VT being successfully induced in the post partum period only following the administration of progesterone supplements.[3] On the other hand, an increase in central sympathetic activity has been reported in normal pregnancy and an increased sensitivity to circulating catecholamines during pregnancy has been proposed as a potential trigger for VT.[2]

Treatment of arrhythmias in pregnancy can pose a challenge since the potential impact of antiarrhythmic drug therapy on the human fetus has not been well studied. The use of beta-blockers is generally considered safe particularly in the later stages of pregnancy although they are associated with fetal bradycardia, neonatal hypoglycemia and intra-uterine growth restriction. Calcium-channel antagonists such as verapamil are also considered safe in the second and third trimester both for inhibition of pre-term labor and for treatment of arrhythmias. However, in our patient neither drug was effective in suppressing her VT and therefore catheter ablation was performed.

Catheter ablation for drug-resistant supraventricular (but not ventricular) arrhythmias complicating pregnancy has been well described in small series and isolated case-reports. In the largest series of nine patients ranging from 12-38 weeks gestation, ablation was performed for a variety of supraventricular arrhythmias.[4] Successful ablation without complication was achieved in all patients using either minimal or zero fluoroscopy. The latter can be facilitated by the use of electroanatomic mapping systems and/or intra-cardiac echocardiography [4] and is attractive in pregnancy to minimise risk to the fetus. Guidelines suggest that after the first trimester there is no evidence of an increased fetal risk of congenital malformations, intellectual disability, growth restriction, or pregnancy loss at doses of radiation to the pregnant woman of <50 mGy.[5]

Recent European guidelines propose that catheter ablation of VT should be considered in pregnancy in the case of drug-refractory and poorly tolerated tachycardia (Class IIb recommendation, level of evidence C).[5] To the best of our knowledge, this is the first report describing successful catheter ablation for new-onset right ventricular outflow tract VT during pregnancy. This arrhythmia is usually well tolerated, is often sensitive to beta-blockers or verapamil and frequently improves or disappears completely during the post-partum period.[2] However, our patient was refractory to antiarrhythmic drug therapy and, owing to increasingly unstable episodes, underwent successful definitive treatment with catheter ablation without complication.
